# Radiofrequency Ablation and Alcohol Neurolysis of the Splanchnic Nerves for a Patient With Abdominal Pain From Pancreatic Cancer

**DOI:** 10.7759/cureus.10758

**Published:** 2020-10-01

**Authors:** Rana AL-Jumah, Ivan Urits, Omar Viswanath, Alan D Kaye, Jamal Hasoon

**Affiliations:** 1 Department of Anesthesia, Baylor College of Medicine, Houston, USA; 2 Department of Anesthesia, Critical Care and Pain Medicine, Beth Israel Deaconess Medical Center – Harvard Medical School, Boston, USA; 3 Pain Management, Valley Pain Consultants, Envision Physician Services, Phoenix, USA; 4 Anesthesiology, Louisiana State University Health Sciences Center, Shreveport, USA

**Keywords:** pancreatic cancer, chronic pain, celiac plexus, splanchnic nerves, cancer pain

## Abstract

Abdominal pain related to gastrointestinal malignancy can be notoriously difficult to manage and can lead to significant morbidity and suffering. The blockade of the celiac plexus has traditionally been performed for alleviating abdominal pain related to malignancy. Visceral structures that are innervated by these nerves include the pancreas, liver, gallbladder, mesentery, omentum, and the gastrointestinal tract from the stomach to the transverse colon. Alternatively, this pain can be treated by disrupting visceral nociceptive signals at the splanchnic nerves. In this report, we describe our experience of treating a 50-year-old male patient suffering from severe abdominal pain related to pancreatic cancer with multiple liver metastases. The patient failed medication management and had an international normalized ratio of 1.6, which was a concern for performing a celiac plexus block given the proximity of major vascular structures. The patient instead underwent radiofrequency ablation (RFA) as well as alcohol neurolysis of the bilateral splanchnic nerves and obtained significant relief from the procedure.

## Introduction

The treatment of abdominal pain related to pancreatic cancer can be very challenging. The origin of pain related to malignancy may be somatic, visceral, or neuropathic. Most of the pain is due to inflammation or tissue damage of the abdominal viscera and may include ductal obstruction. The pain is localized within the upper and middle abdomen by the visceral nociceptive signals traveling along sympathetic fibers to the celiac plexus nerves, which are located around the T12-L2 vertebrae. These signals are then carried to the splanchnic nerves at the T5-T12 dorsal root ganglia [1-4].

Many patients with pancreatic cancer experience intractable abdominal pain that cannot be controlled on non-opioid medications such as non-steroidal anti-inflammatory drugs (NSAIDs) [1-3]. Opioid medications are the mainstay of therapy for cancer-related pain. However, they often offer patients pain relief at the cost of addictive analgesia, sedation, and constipation. These side effects can be detrimental to pancreatic cancer patients already suffering from gastrointestinal dysfunction [2]. If a patient’s pain is not well controlled on oral medications, including opioids, then interventional procedures should be utilized to help with pain control and improvement of the quality of life. A neurolytic celiac plexus block is generally performed for upper abdominal malignancy, such as pancreatic cancer, and is typically the intervention of choice for abdominal malignancy pain in patients with a short survival expectancy. Alternatively, this pain can be treated by disrupting visceral nociceptive signals at the splanchnic nerves.

## Case presentation

Our patient was a 50-year-old male suffering from severe abdominal pain related to pancreatic cancer with multiple liver metastases. His life expectancy was less than six months. He had severe and debilitating abdominal pain that was not relieved with medication management including acetaminophen, NSAIDs, gabapentin, and high-dose opioids. The patient consistently reported a pain level of 10/10 intensity on a numerical rating scale (NRS). Since the patient failed conservative therapy with medication management, he was referred to interventional pain management for a celiac plexus block to better manage his pain.

After reviewing the patient’s medical chart, it was noted that this patient was coagulopathic as his international normalized ratio (INR) had risen from 1.1 to 1.6 over an eight-day period. Since the celiac plexus lies anterior and laterally around the aorta, needles are often placed through the aorta to perform a traditional celiac plexus block. Given the patient’s elevated INR, we decided to target the splanchnic nerves bilaterally instead. Additionally, given that this patient’s INR was continuing to rise, there was concern that he may not be a candidate for future interventions if he continued to become more coagulopathic. For this reason, we decided to perform both radiofrequency ablation (RFA) and alcohol neurolysis of the splanchnic nerves to ensure full effect. The patient was counseled regarding the risks and benefits of these injections and he consented to undergo the procedure.

**Figure 1:**
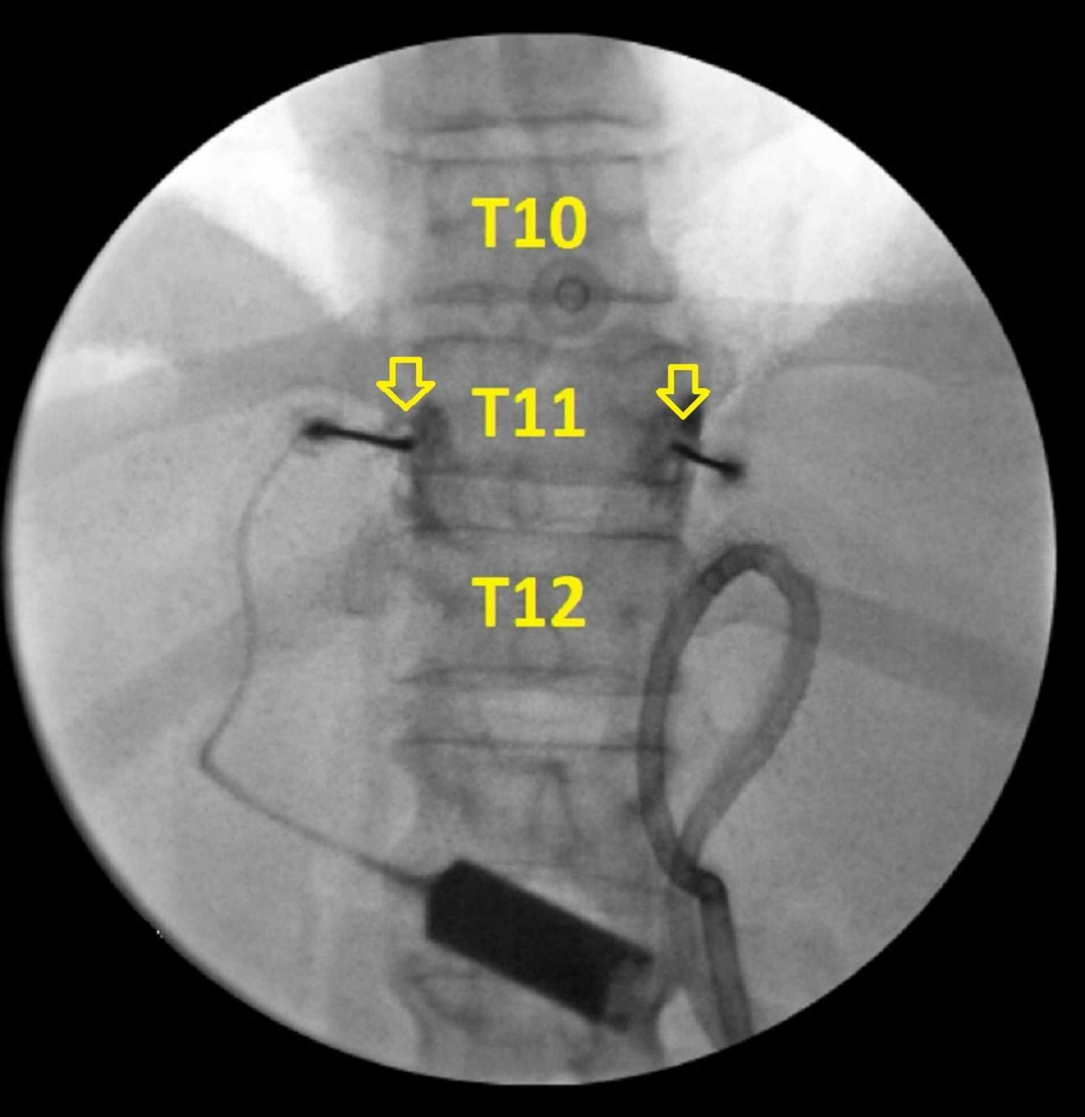
Anterior-posterior view of the procedure at the T11 level This image is an anterior-posterior view of the thoracic spine with fluoroscopy. The needles were placed along the T11 vertebral body, which is labeled in the image. The yellow arrows highlight needle placement along the T11 vertebral body. The needles were placed just laterally to the vertebral body where the splanchnic nerves travel. It is important to note that the needles must be placed carefully to avoid injuring the lungs.

The patient was then taken to a procedure room and placed in the prone position. The thoracolumbar spine was visualized. The skin was anesthetized with 2% lidocaine before introducing the needles used for RFA. The needles were advanced towards the anterolateral aspect of the T11 vertebral body (Figure 1). Once the needles were confirmed to be in proper positioning, sensory testing was performed and caused abdominal stimulation in the same region of his abdominal pain. The nerves were then anesthetized with 2 mL of 2% lidocaine before RFA was performed. RFA was performed at 80 degrees Celsius for 90 seconds on both sides. The needles were withdrawn slightly to create additional lesions along the vertebral body using the same technique.

**Figure 2:**
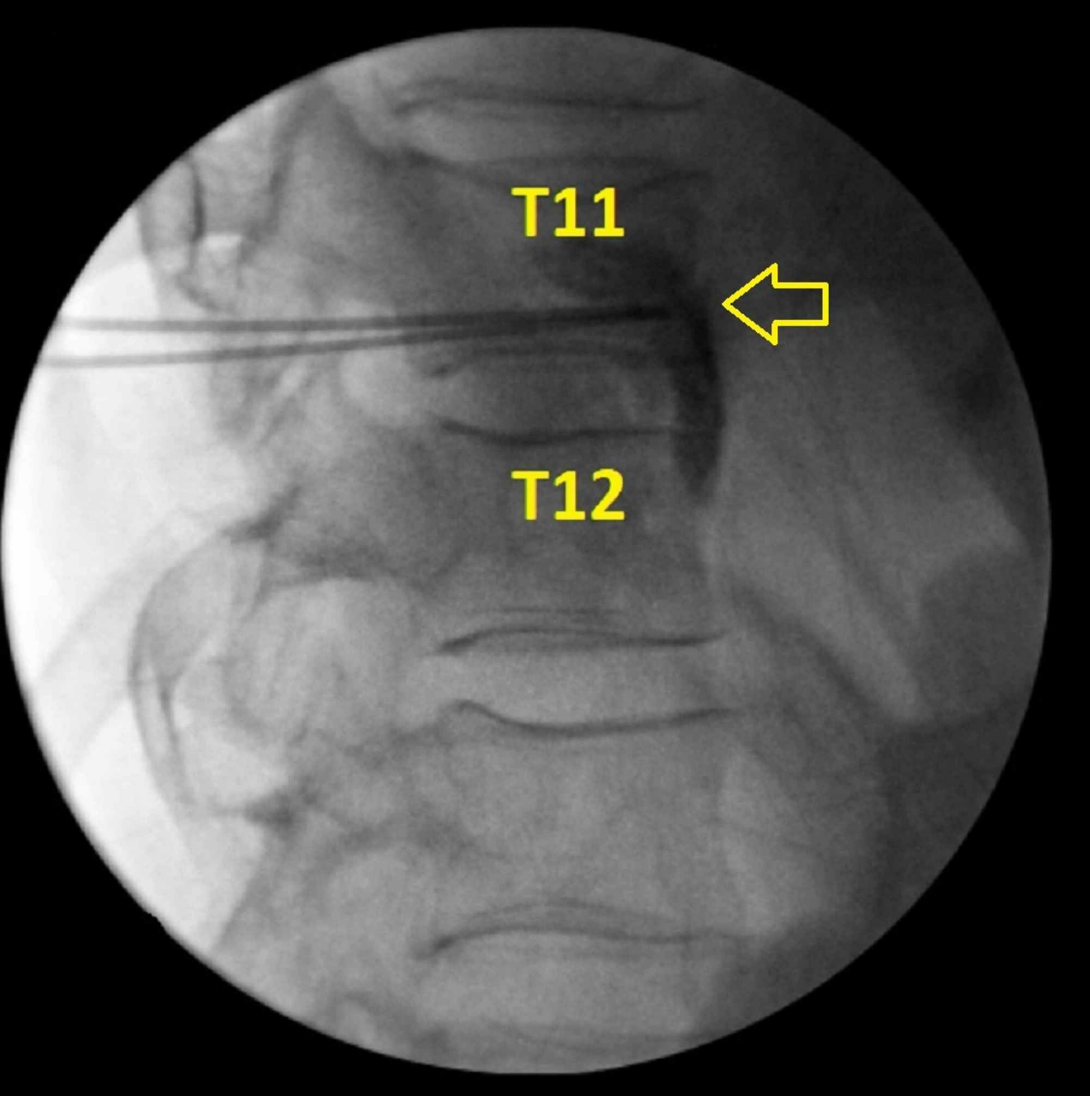
Lateral view of the thoracic spine during the procedure This image is a lateral view of the thoracic spine during the procedure. Radiofrequency ablation was carried out along the lateral wall of the T11 vertebral body, which is labeled in the image. This image also demonstrates contrast spread during alcohol neurolysis. The yellow arrow highlights needle placement along the T11 vertebral body as well as contrast spread during alcohol neurolysis.

Next, the needles were placed near anterolateral aspect of the T11 vertebral body where the splanchnic nerves typically are positioned. Nonionic contrast was then used to verify proper needle placement before performing alcohol neurolysis. After confirming proper needle placement, another 3 mL of 2% lidocaine was administered on each side. Finally, 8 mL of 100% ethyl alcohol mixed with 2 mL of contrast was then divided equally and administered with intermittent fluoroscopy to confirm appropriate spread of the solution (Figure 2).

## Discussion

The patient reported significant improvement in his pain shortly after the procedure, which was related to the local anesthetic that had been injected. The patient reported a pain level of 4/10 on NRS while in the recovery room, which lasted until the next morning. He then had an exacerbation of his pain the following day with a pain score of 8/10 on NRS, which lasted until the fourth post-procedure day. On the fourth post-procedure day, the patient reported progressive improvement of his pain. On the seventh post-procedure day, he reported an 80% improvement in his pain and was requiring significantly less opioids for pain management. The patient experienced sustained relief through the next three weeks of his hospitalization and was discharged to hospice care on minimal opioid requirements.

Due to the location of pancreatic cancer, the celiac plexus and splanchnic nerves are both good targets for providing pain relief. RFA as well as chemical neurolysis of the nerves have been shown to be beneficial as therapeutic measures for patients with chronic abdominal pain due to pancreatic cancer [1,5]. RFA is a technique that achieves thermal coagulation of the nerves by utilizing high frequency alternating current to heat the tissues. The cellular proteins denature as a result of the high heat and can potentially cause nerve destruction [5,6]. A retrospective observational study involving patients with chronic abdominal pain due to malignancy who underwent splanchnic nerve RFA was carried out over a 12-24-month period to assess procedural effectiveness. The study demonstrated that splanchnic nerve RFA resulted in a decrease in pain scores, opiate analgesia use, long-term debilitating chronic pain, as well as increased daily activities [5]. Chemical neurolysis of the celiac plexus or splanchnic nerves can be done with phenol or alcohol to destroy the nerve cells. The alcohol causes sympathetic denervation by inducing inflammation and necrosis on direct contact [7]. The duration of effectiveness in patients receiving neurolysis varies but typically lasts several months [4].

Complications of splanchnic and celiac plexus blocks are rare, but physicians should be aware of common situations. Those that are relatively common include hypotension and diarrhea related to the blockade of sympathetic signals and unopposed parasympathetic response. Physicians should also be aware of the risk of pneumothorax when performing splanchnic nerve blocks. It is very important that needles are kept medially against the vertebral body to reduce the risk of lung injury. With proper technique and preparation, these complications can be anticipated and avoided.

## Conclusions

Treatment of the bilateral splanchnic nerves may provide significant relief from abdominal pain related to pancreatic cancer. Additionally, we propose that interventional pain physicians should consider targeting these nerves when the blockade of the celiac plexus is deemed to be dangerous.
